# Anesthesia Management Using Remimazolam in A Patient With Bernard‐Soulier Syndrome: A Case Report

**DOI:** 10.1002/ccr3.70519

**Published:** 2025-05-16

**Authors:** Tomoharu Shakuo, Yusuke Ishida, Daiki Sugawara, Masato Ryo, Haruko Okazaki, Katsunori Oe

**Affiliations:** ^1^ Department of Anesthesiology Showa Medical University School of Medicine Tokyo Japan; ^2^ Department of Anesthesiology Showa Medical University Northern Yokohama Hospital Yokohama Japan; ^3^ Division of Medical and Dental Cooperative Dentistry, Department of Perioperative Medicine Showa Medical University School of Dentistry Tokyo Japan; ^4^ Department of Anesthesiology St. Luke's International Hospital Tokyo Japan

**Keywords:** Bernard‐Soulier syndrome (BSS), remimazolam, thrombocytopenia, thromboelastography, viscoelastic test

## Abstract

Bernard‐Soulier syndrome (BSS) is a platelet dysfunction disorder characterized by massive thrombocytopenia and a lack of platelet aggregation. Remimazolam, a short‐acting benzodiazepine sedative, is believed to have minimal effects on platelets. Here, we report the safe use of remimazolam for anesthesia management in a patient with BSS.

**Abbreviations**
BIS: bispectral Index
BSS: Bernard‐Soulier syndrome
CRT: clot reaction time
GP: glycoprotein
Hb: hemoglobin
MA of CRT: maximum amplitude of CRT
NSAIDs: non‐steroidal anti‐inflammatory drugs
POD: postoperative day
PT‐INR: Prothrombin Time‐International Normalized Ratio
TEG: thromboelastograph

## 1 Introduction

Bernard‐Soulier syndrome (BSS) is a congenital platelet function disorder characterized by a tendency to bleed, thrombocytopenia with giant platelets, and a lack of platelet aggregation due to deficiency in the platelet membrane glycoprotein (GP) Ib/IX complex [1]. Patients with BSS undergoing surgical procedures have a potential risk of difficulty in achieving hemostasis, making careful consideration of anesthesia management essential [2]. When selecting anesthetic agents, it is important to choose those that do not significantly impair platelet function. In 2020, remimazolam, a short‐acting benzodiazepine, became available for use in general anesthesia in Japan [3], and its application in various cases has been increasingly reported. Being a benzodiazepine, remimazolam is thought to have minimal effects on platelet function [4]. In this report, we present the anesthesia management of a patient with BSS undergoing surgery for nasal septum deviation using remimazolam, where the patient was managed without the occurrence of severe bleeding complications during or after surgery. Written, informed consent was obtained from the patient for publication of this case report.

## 2 Case History/Examination

The patient was a 31‐year‐old male, 181.0 cm tall, weighing 88.8 kg. He had a history of frequent nasal obstruction and nasal discharge, and was scheduled for surgery at our hospital after being diagnosed with nasal septum deviation. The patient had a history of unexplained thrombocytopenia since childhood, and had been diagnosed with BSS approximately 2 months earlier following detailed investigation. Preoperative laboratory tests revealed a platelet count of 51,000/μL, hemoglobin (Hb) of 13.3 g/dL, and Prothrombin Time‐International Normalized Ratio (PT‐INR) of 1.10. In accordance with his BSS diagnosis, his platelet count was low, and his platelets lacked hemostatic function. Therefore, a conference involving otolaryngologists, anesthesiologists, and hematologists was conducted, and a decision to administer platelet transfusions preoperatively, intraoperatively, and postoperatively was made. For the anesthesia management, it was decided to select drugs that would minimally impair platelet function. Hence, remimazolam, a benzodiazepine thought to have little impact on platelet function, was selected. Anesthesia management was also guided by the evaluation of platelet function using a Thromboelastograph (TEG) 6 s (TEG 6 s: Haemonetics Corp., Boston, MA, USA) blood viscoelastic testing device during surgery. A preoperative blood test just before the surgery showed a platelet count of 30,000/μL. Upon entering the operating room, the patient's blood pressure was 150/77 mmHg, heart rate was 69 beats/min, and SpO_2_ was 98%.

## 3 Methods (Differential Diagnosis, Investigations, and Treatment)

**FIGURE 1:**
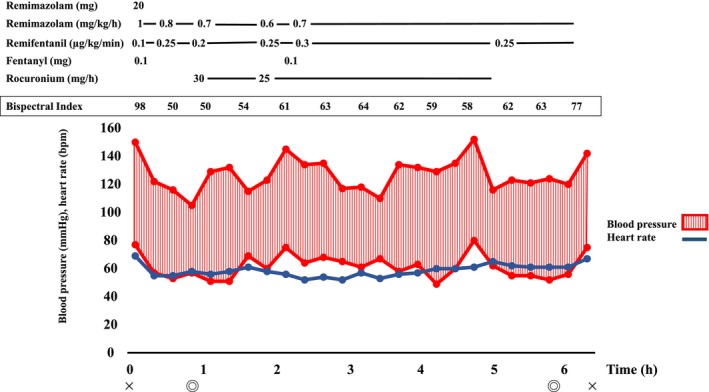
Patient's anesthesia record. Cardiovascular dynamics were stable during anesthesia management with remimazolam. BIS was intraoperatively maintained between 40 and 65, indicating adequate anesthesia depth.

**FIGURE 2:**
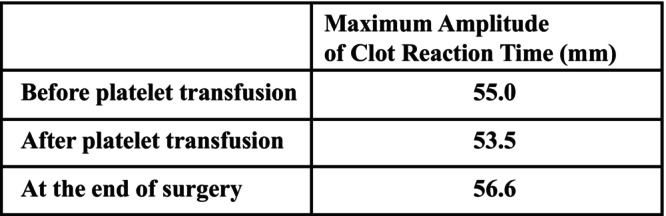
Intraoperative course of blood viscoelasticity tests. The maximum amplitude of clot reaction time remained almost in the normal range.

Anesthesia was induced with 20 mg remimazolam, 100 μg fentanyl, remifentanil at 0.1 μg/kg/min, and 90 mg rocuronium. Anesthesia was maintained with infusions of remimazolam at 0.7–1.0 mg/kg/h, remifentanil at 0.1–0.3 μg/kg/min, and rocuronium at 25–30 mg/h. Intubation was performed gently using an 8 mm endotracheal tube to avoid bleeding. After anesthesia induction, 10 units of platelet concentrate were transfused, and 1000 mg of tranexamic acid was administered before the commencement of surgery to prevent bleeding. Intraoperative monitoring included invasive arterial blood pressure measurement, SpO_2_, and bispectral index (BIS) (Figure 1). During the operation, an additional 10 units of platelet concentrate were transfused. Blood viscoelastic testing was also performed as needed (Figure 2). The maximum amplitude of clot reaction time (MA of CRT) remained within the normal range, fluctuating between 53.5 and 56.6 mm. Fentanyl was administered during the operation for postoperative pain relief. Non‐steroidal anti‐inflammatory drugs (NSAIDs), which might exacerbate bleeding, were avoided. The total anesthesia time was 6 h and 12 min, surgery lasted 5 h and 5 min, the total infusion volume was 2500 mL, 20 units of platelets were transfused, urine output was 2050 mL, and blood loss was 200 mL. After the operation, anesthesia was discontinued, and the patient was extubated once he was fully awake and able to follow commands. The patient remained stable without bleeding post‐extubation, and was transferred to the ward. Postoperatively, his Hb was 11.2 g/dL, and platelet count was 50,000/μL. On postoperative day (POD) 1, 10 units of platelets were transfused as a precaution. The patient had an uneventful postoperative course without severe bleeding complications, and was discharged on POD 10.

## 4 Conclusion and Results

Based on our experience with this case, the use of remimazolam appears to be effective when managing anesthesia in BSS patients with platelet function abnormalities. In conclusion, we successfully used remimazolam as a sedative‐hypnotic during general anesthesia in a patient with thrombocytopenia due to BSS. Future large‐scale studies to evaluate the impact of remimazolam on platelet function would be useful.

## 5 Discussion

BSS is a congenital disorder characterized by impaired platelet aggregation due to a deficiency (quantitative abnormality) or dysfunction of the GPIb/IX/V complex [1]. The GPIb/IX/V complex acts as a receptor for von Willebrand factor. In BSS patients, platelet aggregation tests show aggregation with many agonists, such as ADP, collagen, epinephrine, and thrombin, although aggregation in response to ristocetin is absent. This disorder follows an autosomal recessive inheritance pattern [1]. Clinically, patients present with petechiae, purpura, epistaxis, gingival bleeding, and gastrointestinal bleeding [1]. In rare cases, patients might experience severe bleeding during injuries or surgeries [2]. The severity of these bleeding symptoms varies among patients, ranging from mild to life‐threatening, and it is also reported that the condition can worsen during adolescence or adulthood [5]. Given these factors, patients with BSS who undergo surgery might face significant challenges in relation to hemostasis during the procedure. Additionally, head and neck surgeries carry the risk of bleeding into the airway, with potentially fatal complications, such as asphyxia. Therefore, meticulous hemostatic strategies are required from the preoperative period through the perioperative management.

Inhalational anesthetics, which are commonly used as sedatives in anesthesia management, are known to reduce platelet function to some extent [6]. Sevoflurane, for example, inhibits the formation of thromboxane A2 by suppressing cyclooxygenase activity, thereby reducing platelet aggregation [7]. In this case, we opted to maintain anesthesia without the use of inhalational anesthetics. However, it has also been reported that the intravenous anesthetic, propofol, can decrease platelet aggregation [6]. On the other hand, midazolam, a benzodiazepine, is known to have minimal impact on platelets [8]. Remimazolam, an intravenous anesthetic that became available in Japan in 2020, is a benzodiazepine with a shorter half‐life compared to traditional benzodiazepines and has the advantage of an available antagonist [3]. It also has minimal effects on hemodynamics [9]. Furthermore, being a benzodiazepine, remimazolam is likely to have minimal impact on platelet function. Thus, anesthesia management with remimazolam might reduce the risk of bleeding in patients with impaired platelet function or low platelet counts. For analgesia, we used fentanyl and remifentanil, opioids known to have minimal effects on platelet function [4, 10].

Additionally, in this case, we managed the patient using TEG to assess blood viscoelasticity. TEG allows for comprehensive evaluation of coagulation parameters in a short time, making it useful for diagnosing bleeding disorders and assessing the efficacy of treatment [11, 12]. In TEG, the CRT measures the overall time required for clot formation, which is used to evaluate platelet function and the efficiency of coagulation factors. In this case, the MA of CRT remained within the normal range, suggesting that platelet function was maintained. TEG‐guided management has been shown to reduce the amount of blood product transfused and associated complications [13]. Karataş et al. reported successful hemostatic management using TEG in BSS patients undergoing anesthesia [14]. In this case, no further decrease in platelet function was observed with TEG, indicating that management with remimazolam was effective for this high‐risk patient. Additionally, use of tranexamic acid and desmopressin has been reported for hemostatic control in the perioperative management of BSS patients [15, 16]. In this case, tranexamic acid was administered after the induction of anesthesia. Intraoperatively, platelet transfusions were also performed as part of the hemostatic strategy. Furthermore, we avoided the use of drugs that could potentially increase the risk of bleeding, such as NSAIDs, during the perioperative period [17].

In anesthesia management, maintaining an appropriate dosage of remimazolam is crucial. It is advisable to adjust the sedative dosage as needed during surgery by using electroencephalography monitors, such as BIS (Covidien, Boulder, CO, USA) or Sedline (Masimo, Irvine, CA, USA) [18]. In this case, anesthesia was managed while monitoring BIS values, with no complications such as intraoperative awareness. A PubMed search revealed that there have been no case reports describing the use of remimazolam for the anesthesia management of BSS patients.

## Author Contributions

**Tomoharu Shakuo:** conceptualization, writing – original draft. **Yusuke Ishida:** conceptualization, writing – original draft, writing – review and editing. **Daiki Sugawara:** writing – review and editing. **Masato Ryo:** writing – review and editing. **Haruko Okazaki:** writing – review and editing. **Katsunori Oe:** writing – review and editing.

## Ethics Statement

The authors have nothing to report.

## Consent

Written, informed consent was obtained from the patient for publication of this case report and the accompanying images.

## Conflicts of Interest

The authors declare no conflicts of interest.

## Data Availability

The data that support the findings of this study are available from the corresponding author upon reasonable request.
